# Exploring the various effects of Cu doping in hydroxyapatite nanoparticle

**DOI:** 10.1038/s41598-024-53704-x

**Published:** 2024-02-10

**Authors:** Alireza Noori, Mahdieh Hoseinpour, Sedighe Kolivand, Nasrin Lotfibakhshaiesh, Somayeh Ebrahimi‐Barough, Jafar Ai, Mahmoud Azami

**Affiliations:** 1 Department of Tissue Engineering and Applied Cell Sciences, School of Medicine, Semnan University of Medical Sciences, Semnan, Iran; 2 Department of Tissue Engineering, School of Advanced Technologies in Medicine, Tehran University of Medical Sciences, Tehran, Iran; 3 Recombinant Proteins Department, Breast Cancer Research Center, Motamed Cancer Institute, ACWCR, Tehran, Iran

**Keywords:** Nanoscale biophysics, Biophysical chemistry, Biomaterials, Ceramics

## Abstract

Adding foreign ions to hydroxyapatite (HAp) is a popular approach for improving its properties. This study focuses on the effects of calcium substitution with copper in HAp. Instead of calcium, copper ions were doped into the structure of hydroxyapatite nanoparticles at 1%, 3%, and 5% concentrations. XRD analysis showed that the amount of substituted copper was less than needed to generate a distinct phase, yet its lattice parameters and crystallinity slightly decreased. Further, the results of degradation tests revealed that copper doping in hydroxyapatite doubled calcium ion release in water. The incorporation of copper into the apatite structure also boosted the HAp zeta potential and FBS protein adsorption onto powders. According to antibacterial investigations, a concentration of 200 mg/ml of hydroxyapatite containing 5% copper was sufficient to effectively eradicate E. coli and S. aureus bacteria. Furthermore, copper improved hydroxyapatite biocompatibility. Alkaline phosphatase activity and alizarin red tests showed that copper in hydroxyapatite did not inhibit stem cell differentiation into osteoblasts. Also, the scratch test demonstrated that copper-containing hydroxyapatite extract increased HUVEC cell migration. Overall, our findings demonstrated the utility of incorporating copper into the structure of hydroxyapatite from several perspectives, including the induction of antibacterial characteristics, biocompatibility, and angiogenesis.

## Introduction

Hydroxyapatite nanoparticles with the chemical formula Ca_10_(PO_4_)_6_(OH)_2_ are of great interest in bone tissue engineering because they constitute a mineral component of bone. The hydroxyapatite structure allows for the inclusion of different ions, thereby potentially augmenting its biocompatibility, bioactivity, and mechanical characteristics^1,2^. Researchers have tried substituting Zn, Sr, Ba, Cu, Pb, Mg, Na, and K for calcium, CO_3_, As, S, Si, Ge, Cr, and B for phosphorus, and CO_3_, O, F, Cl, and Br for hydroxyl to improve hydroxyapatite properties^1,2^.

Copper is among the metallic elements that can be incorporated into the hydroxyapatite structure. Being a necessary mineral for human health, copper deficiency can cause anemia, pancytopenia, and neurodegeneration, among other complications^3–5^. Copper also play a crucial role in the skeletal system. It is involved in the first step of forming stable collagen fibers, which is commonly attributed to the action of lysyl oxidase, an enzyme that cross-links collagen fibers and requires copper as a cofactor^6,7^. In addition, research indicates that copper deficiency may contribute to the development of brittle bones, but this can be remedied by consuming copper supplements^6^. Further, copper acts as a cofactor for superoxide dismutase, which helps eliminate free radicals produced during bone resorption^7^. Copper can also contribute to angiogenesis^8^. One method for promoting angiogenesis involves inducing hypoxia and upregulating the expression of hypoxia-inducible factor 1α (HIF-1α), which can be triggered by copper ions. Sen et al. have revealed that copper promotes wound healing by boosting vascular endothelial growth factor (VEGF) expression in endothelial cells^9^. In addition, when present in the proper concentration, copper encourages the proliferation of endothelial cells^10^.

On the other hand, the gradual release of copper at the appropriate concentration can kill bacteria. Copper's antibacterial impact is thought to be caused by multiple mechanisms, including the disruption of bacterial membrane integrity, the generation of free radicals, and the alteration of bacterial protein and enzyme structure^11^. It is critical to remember that an increased concentration of copper may cause toxicity in human cells; thus, the concentration of copper must be optimized to precisely target bacteria while not hurting human cells^12^. In this context, Chengtie Wu and colleagues found that adding copper ions to bioactive glass increased the expression of genes involved in osteogenesis and blood vessel formation. Copper also made bioactive glass efficient against E. coli. It is imperative to highlight that a high concentration of copper (derived from 100 mg/ml of a 5% wt copper-doped sample) was also toxic to human cells^13^.

The aim of this article was to investigate the multiple benefits of doping copper into hydroxyapatite's structure. Several techniques were used to assess how copper's presence affected the physicochemical characteristics of hydroxyapatite nanoparticles. Special attention was given to the nanoparticles' degradation, ion release, and protein absorption capacity, which have potential applications in drug delivery studies. The powders' ability to eradicate both gram-positive and gram-negative bacteria was also examined. The effect of copper doping in HAp on osteogenic differentiation of rat mesenchymal stem cells (rMSCs) was investigated using alkaline phosphatase (ALP) activity and alizarin red staining. Additionally, the angiogenic potential was assessed using the human umbilical vein endothelial cells (HUVECs) cell migration test.

## Materials and methods

The present study focuses on investigating the effects of copper (Cu) doping in hydroxyapatite nanoparticles. It has been approved by the ethics committee of Tehran University of Medical Sciences (IR. TUMS. VCR. REC. 1397. 911) and follows the guidelines and regulations set by the editorial and publishing policies of the scientific reports journal**.** All methods were performed in accordance with the relevant guidelines and regulations.

### Synthesis of hydroxyapatite nanoparticles

The hydroxyapatite used in this study was synthesized using the co-precipitation technique^14^. Initially, 0.1 mol of calcium nitrate tetrahydrate (Merck, Germany) and 0.06 mol of diammonium hydrogen phosphate (Merck, Germany) were dissolved individually in deionized water, and the pH of both solutions was adjusted to 10 with ammonia. After one hour of agitation, the phosphate solution was added to the calcium nitrate solution drop by drop, while the pH of the solution was kept in the range of 10. The milky solution was stirred on a stirrer for 24 h and then aged for 48 h at room temperature to obtain particulates with a uniform morphology. The solution was then centrifuged to separate the supernatant water from the apatite gel, and the gel was washed three times with distilled water to eliminate any leftover ammonia and nitrate. The obtained gel was dried in an oven at 120 °C for two days before being pulverized in a mortar. The synthesis of copper-containing hydroxyapatites was conducted using the aforementioned procedure, with the exception that copper nitrate hexahydrate (Merck, Germany), at predetermined concentrations, was dissolved in 20 ml of deionized water and added to the solution containing calcium nitrate after adjusting its pH to 10. The remaining stages were carried out identically to the pure hydroxyapatite sample synthesis. Copper-substituted hydroxyapatite powders were prepared using three different starting concentrations of copper nitrate solution. These powders, labeled Cu-HA 1%, Cu-HA 2%, and Cu-HA 5%, contained 1, 2, and 5 mol% Cu, which replaced Ca in the hydroxyapatite structure, respectively. To investigate the effects of heating on the morphology and crystal structure of HA, the dried HA powders were further calcined at 850 °C for 3 h.

### Physicochemical characterization of hydroxyapatite nanoparticles

X-ray fluorescence analysis (XRF) (Zetium, Panalytical, UK) was used to determine the types and quantities of chemical components present in the samples.

X-ray diffraction (Bruker, D8 Advance X-ray Diffraction (XRD) spectrophotometer, Germany) was used to find out the phase and crystallographic features of both uncalcined and calcined samples. It was performed under ambient conditions using CuK as the radiation source over the angle range 10–80, with a step size of 0.02° and a scan speed of 0.05°/second. X’Pert HighScore Plus (v. 2.1.0, PANalytical B.V. Almelo, The Netherlands) was used for XRD patterns analysis. The generated spectra were compared with the peaks found in the software's joint committee on powder diffraction standards (JCPDS) cards. Each sample's lattice parameters and crystal size were refined by the Material Analysis Using Diffraction (MAUD) program. The crystallinity index was determined using OriginLab (v. 2018 SR1 Academic, OriginLab Corporation, Northampton, MA, USA) software, which involved dividing the crystalline area of the peaks by the overall area of the peaks.

The FTIR spectra of the powders were recorded using the Perkin-Elmer FTIR spectrometer to identify the functional groups of the synthesized powders. Using the KBr pellet technique, the transmission FTIR spectrum was collected from 400 to 4000 cm^−1^.

A zeta potential analyzer (Malvern Instruments, UK) was used to measure the zeta potential of HAp particles. At a pH of 7.0 and a temperature of 25 °C, the measurement was carried out in a 10 mM NaCl solution.

Field emission scanning electron microscopy (FESEM) (TESCAN MIRA3, Czech Republic) was used to examine the morphologies of the prepared samples. The particle size and distribution were determined by analyzing FESEM micrographs using ImageJ (v. 1.51j8, Wayne Rasband, National Institutes of Health and University of Wisconsin, Bethesda, MD, USA) software.

To analyze the effect of copper incorporation into the HAp structure on powder degradation, pH change of the culture medium, which is influenced by the ions released in the culture medium, was evaluated. To do this, 75 mg of the powders were carefully dispensed into the individual wells of a 24-well plate, and 200 µl of the culture medium were poured over them. The plate was then placed in a 37 °C incubator. After adding the culture medium to the powders, the pH of the medium was determined at various time intervals (0.5, 1, 2, 3, 6, 24, and 72 h).

To further understand how the powders degrade, the concentrations of calcium and copper ions released from the powders in the aqueous media were measured using specialist kits. 250 µl of distilled water were added to 2 ml microtubes containing 30 mg of powder. At predetermined intervals, water was taken from the samples, emptied into a separate container, and refrigerated until the elemental content was measured. Following this, an equivalent volume of 250 µl of fresh distilled water was added to each sample. This procedure was then repeated every few days until the measurement period concluded. Both calcium and copper concentration measurement kits were provided by Derman Faraz Kaveh Company, Isfahan, Iran. The measuring procedure was followed as specified by the manufacturer.

The concentration of absorbed proteins in the powders was determined using the Bradford method. 75 mg of different powders were poured into 2 ml microtubes, and 300 µl of culture media containing 50% fetal bovine serum (FBS) were added to the microtubes. The mixture was incubated for 4 h to allow the proteins to bind to the powders. After that, the medium was removed, and the powders were rinsed once with water to eliminate the loosely attached proteins. The particles were then treated with 200 µl of a 6 M urea solution (Merck, Germany)^15^. The mixture was left to sit for one day, allowing the bound proteins to separate and enter the liquid. The Bradford assay procedure was performed according to the supplied instructions provided by the kit’s creator (Arsam Fara Zist, Uraemia, Iran).

### Antibacterial studies

To test the copper-containing hydroxyapatite's antibacterial effectiveness, E. coli and S. aureus bacteria were used. Hydroxyapatite powders containing 0, 1, 2, and 5% copper were weighed (40, 20, and 10 mg of each powder) and sterilized in an autoclave prior to being put into microtubes. Each microtube received 200 µl of E. coli or S. aureus bacteria at a concentration of 2 × 10^5^ bacteria per milliliter, and the microtubes were then incubated for 24 h at 37 °C. After 24 h, 50 µl of the microtubes medium were withdrawn and evenly disseminated across the surface of the solid agar plates, and the plates were incubated for another 24 h at 37 °C. After 24 h, the plates were finally photographed with a digital camera.

### Cell culture studies

#### Powders’ extract preparation

The dissolution extracts were made according to a methodology adapted from ISO10993-1's standard procedure, as documented in the literature^16^. In a nutshell, 400 mg/mL of hydroxyapatite particles were soaked in serum-free Dulbecco's Modified Eagle's Medium (DMEM; Bioidea, Iran). The mixture was incubated at 37 °C for 24 h, after which the supernatant was centrifuged and collected. After being filtered through a 0.22 µm Millipore membrane, the supernatant was serially diluted (to 200, 100, 50, and 25 mg/mL) in serum-free DMEM.

#### Cell culture

Following standard protocols^17^, rMSCs were isolated from the femurs of 4-week-old rats with no abnormalities. The procedures were authorized by the Ethics Committee of Tehran University of Medical Sciences regarding to ARRIVE guidelines. Briefly, after sacrificing the rats (2 Wistar rats) by administering an overdose of ketamine (100 mg/kg) and xylazine (75 mg/kg), the skin was immediately separated, the femur was severed with scissors, and the surrounding connective tissues were carefully removed. The medullary cavity was exposed by making incisions at the ends of the long bones. Using an 18-gauge syringe, the medulla was carefully flushed with 3 ml of DMEM. To obtain a single-cell suspension, the medium and cells were gently aspirated and expelled using the same needle and syringe. The bone marrow suspensions were cultured in polystyrene flasks for two days, and non-adherent cells were eliminated by washing with PBS and changing the media. The adherent cells were subsequently cultivated in 5% CO_2_/95% air monolayers at 37 °C, with medium replacement occurring twice a week. We used DMEM supplemented with 10% FBS (Bioidea, Iran) and 1% penicillin/streptomycin (Sigma, USA) for cell culture. The second through fifth cell passages were used in subsequent investigations.

The HUVECs were obtained from Iran's Pasteur Institute. They were grown in DMEM supplemented with 10% FBS and 1% (v/v) penicillin and streptomycin. In a 5% CO_2_ incubator, the cells were kept at 37 °C.

#### Cell viability

A toxicity assay on rMSCs and HUVECs was conducted to evaluate the biocompatibility of hydroxyapatite powders. A quantity of 5 × 10^4^ cells was introduced into each well of a 96-well plate. After 24 h, the cells were exposed to extracts obtained from hydroxyapatite powders. The concentrations of the hydroxyapatite powders in the each sample were 400, 200, 100, 50, and 25 mg/ml. After three days of incubation, the morphology of the cells was examined using an inverted-phase microscope (Olympus, Japan).

#### ALP activity

In 48-well plates, rMSCs were grown at a density of 4 × 10^4^ cells per well. As described in the section on the cell viability assay, the culture medium was replenished after 24 h in both the control and test groups. The medium was withdrawn on day 7, and the cells were washed twice with PBS. Subsequently, they were lysed in 200 μL of 0.2% TritonX-100 and frozen/thawed three times at − 20/37 °C. The cell lysates were then centrifuged at 3000 rpm for 30 min, and 50 μL of the supernatant was then carefully transferred to a fresh 48-well plate and well mixed with 200 μL of *p*-nitrophenyl phosphate solution, per the manufacturer's instructions (Darman Faraz Kave, Isfahan, Iran). The OD at 405 nm was determined using the microplate reader.

#### Alizarin red S staining

rMSCs were distributed at a density of 6 × 10^4^ cells per well in 24-well plates. The medium was substituted with the control and treatment groups after two days, as outlined in the cell viability assay. After 14 days, the cells were rinsed with PBS and fixed for 1 h in 4% formaldehyde. The cells were then rinsed once more and exposed to 2% alizarin red (Sigma-Aldrich, USA) for 40 min. After that, the cells were carefully washed with distilled water before being viewed under the inverted microscope.

#### Cell migration assay

The scratch wound test was used to assess HUVEC migration. HUVECs were seeded in 6-well plates at a density of 1 × 10^5^ cells per well, and "scratches" were generated by scraping the monolayer with pipet tips once the cells had attained 80% confluence. Detachable cells were removed with a PBS wash, and thereafter, the cells were handled as specified in the section on cell viability assays. At 0 and 48 h post-treatment, damaged areas were observed under the microscope. ImageJ was utilized for quantifying cell migration. The proportion of the original wounded region (time 0) colonized by endothelial cells was used to calculate the migration of HUVECs into the cell-free area.

### Statistic

For each test, at least three independent experiments were carried out, and the results were provided as the mean ± standard deviation. A one-way ANOVA followed by Tukey's post hoc test was used to undertake statistical analysis of multiple comparisons. A difference was deemed significant if the value of p was less than 0.05.

### Ethics approval and consent to participate

Our project was approved by the Tehran University of Medical Sciences (IR. TUMS. VCR. REC. 1397. 911).

## Results and discussion

### Physicochemical characterization of hydroxyapatite nanoparticles

We used the wet chemical precipitation method to produce copper-doped hydroxyapatite. This method is commonly used for synthesizing doped hydroxyapatite due to its benefits of ultrahigh purity, simplicity, and the ability to achieve nanoscale size^18^.

Table 1 depicts the XRF results. Our preliminary studies showed that precursors and materials utilized in powder synthesis must be carefully selected because the inclusion of any unwanted elements can change the study's outcomes. For instance, if sodium hydroxide is used instead of ammonia to adjust the precursor pH, the resulting hydroxyapatite powder will have a high sodium content. Table 1 demonstrates that cations-to-phosphorus ratios in different samples were close to hydroxyapatite's stoichiometric ratio (1.67). Nevertheless, the quantity of copper that actually entered the apatite structure differed from the amount of copper projected to enter. Moseke et al. also reported that only a minor proportion of the Cu ions present in the initial solution were securely adsorbed to the precipitated hydroxyapatite^19^. Nonetheless, introducing these amounts of copper had a noticeable impact on the apatite structure.

**Table 1:** The HA particle's elemental composition was determined using XRF analysis.

Element	CaO (wt%)	P_2_O_5_ (wt%)	Cu (wt%)	LOI (wt%)	Total (wt%)	Cu/Ca(molar ratio) (%)	(Ca + Cu)/P (molar ratio)
Sample							
HA	51.72	40.28	–	8	100	–	1.66
Cu-HA 1%	51.10	36.77	0.13	12	100	0.22	1.74
Cu-HA 2%	50.42	37.19	0.38	12	100	0.72	1.67
Cu-HA 5%	46.92	36.3	0.78	14	100	1.85	1.59

**Figure 1:**
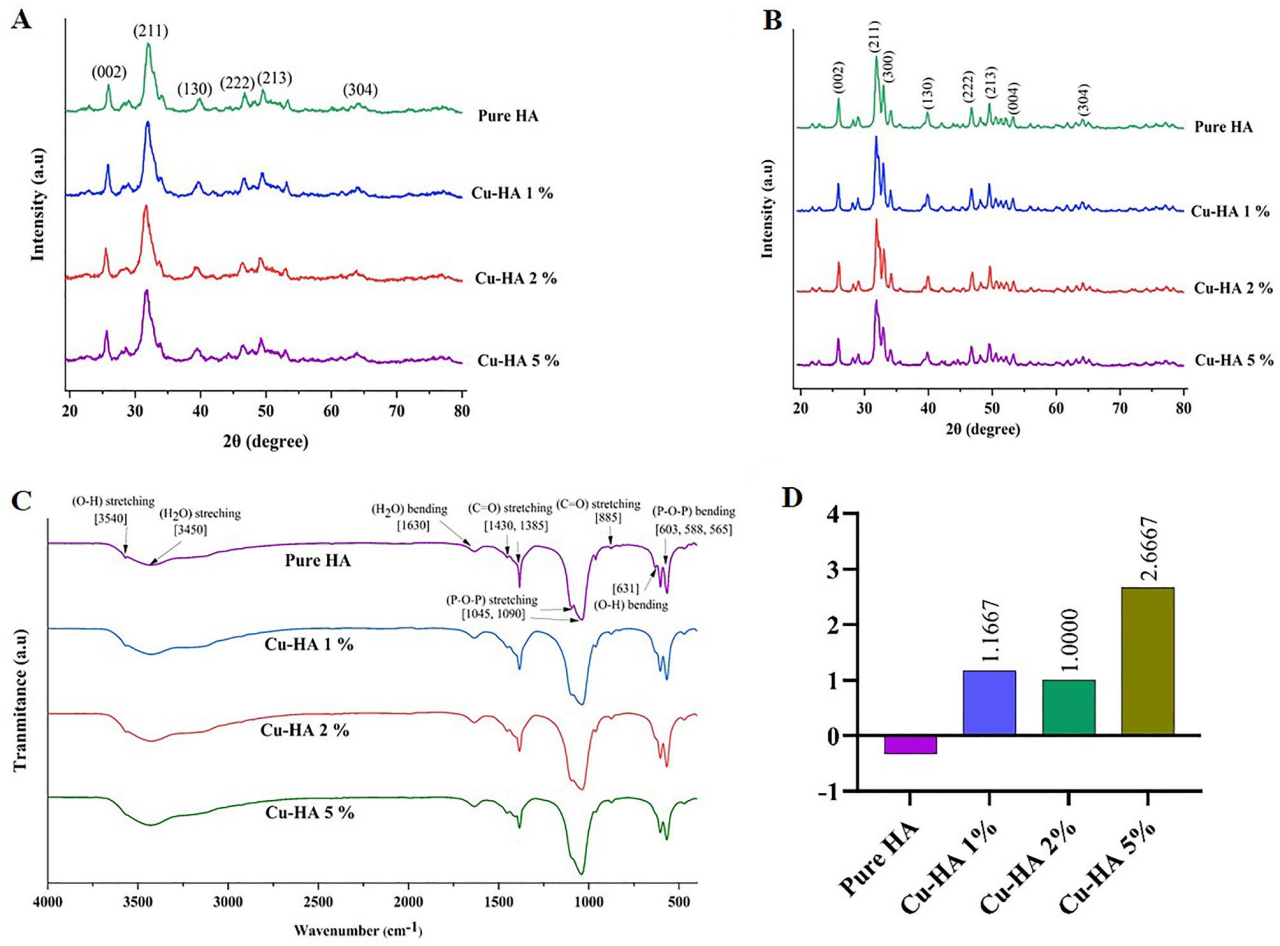
Characterization of hydroxyapatite powders. The XRD patterns of as-dried (**A**) and calcined powders (**B**). FTIR spectra (**C**) and Zeta potential (**D**) of as-dried copper-doped hydroxyapatite.

Figure 1A shows XRD analysis graphs of as-dried hydroxyapatite nanoparticles with varying copper concentrations. Copper inclusion in the hydroxyapatite structure slightly changed peak intensities and angles. Most of these alterations, however, were slight since just a small amount of copper was added to the hydroxyapatite structure. Analysis using Xpert HighScore Plus software confirmed that the addition of copper did not introduce any new phases and that hydroxyapatite remained the sole phase present in the nanoparticles, as evidenced by a match with JCPDS 9-432.

Figure 1B displays the powder XRD patterns of the powders after 3 h of calcination at 850 °C. According to Xpert HighScore Plus software analysis of the peaks, the particles' only phase was hydroxyapatite. Neither the calcination temperature nor the powder's copper content resulted in the formation of a new phase. Comparing the two figures demonstrates that calcination of the powders at 850 °C increased the number and sharpness of the peaks, indicating greater crystallinity. This pattern appears in all samples, independent of copper ion concentration.

We used MAUD to quantitatively evaluate XRD graphs after identifying the material's constituent phase. When particles were not calcined, the concentration of copper ions doped to the HAp structure controlled the change in network parameters (Table 2). Both lattice parameters increased in the Cu-HA 1% sample but decreased rapidly as the copper concentration further increased. The data also shows that calcination temperature had a greater impact on network parameters than copper content. Calcination at 850 °C increased the network parameters in all samples. Compared to non-sintered samples, pure hydroxyapatite had the highest network parameters following calcination. This means that copper inclusion in apatite reduced both lattice parameters, and the magnitude of the loss increased with copper ion concentration.

**Table 2:** Crystallite size, crystallinity, and lattice parameters of copper substituted hydroxyapatite.

Sample	Crystallite size (nm)	Crystallinity (%)	Lattice constant a,c (Å)
As-dried HA	168	60.5	9.4123, 6.8788
As-dried Cu-HA 1%	163	59	9.4248, 6.8813
As-dried Cu-HA 2%	171	55	9.4037, 6.8703
As-dried Cu-HA 5%	165	53.5	9.3865, 6.8561
Calcined HA	403	82	9.4349, 6.8945
Calcined Cu-HA 1%	409	84	9.4287, 6.8909
Calcined Cu-HA 2%	410	78	9.4197, 6.8831
Calcined Cu-HA 5%	351	76	9.4213, 6.8796

Table 2 also reveals how crystallinity varies with copper content and calcination. Copper doping in the apatite structure reduced crystallinity, so the Cu-HA 5% sample had the lowest crystallinity both before and after calcination. On the other hand, heating powders in the furnace increased crystallinity from 60.5 to 82.0% for the pure HA and from 53.5 to 76.0% for the Cu-HA 5% sample. This suggests that heating affected apatite crystallinity more than copper doping. In other words, following calcination, the crystallinity of the hydroxyapatite with the highest copper content was significantly higher than that of pure hydroxyapatite before heating.

The findings presented above are consistent with those of other studies. For instance, Poovendran et al. used a co-precipitation approach to synthesize Cu-doped HAp with varying Ca (10.67–29.2%) and Cu (0.09–0.58%) ratios. The study showed that the introduction of Cu^2+^ led to a reduction in the crystallite and particle size of HAp^20^. Shu et al. replaced up to 25% of hydroxyapatite calcium with copper^21^. Even at 8% copper, hydroxyapatite was the dominant phase, but when the copper concentration grew to 16%, new peaks appeared, indicating that some of the apatite phase was transformed into the Ca_19_Cu_2_(PO_4_)_14_ phase. At 20% copper doping, the secondary phase replaced the apatite phase. Interestingly, at the concentration where a single-phase product occurred, copper reduced the hydroxyapatite crystal lattice dimensions. Furthermore, the crystallinity decreased from 64% in the pure hydroxyapatite to 31% in the 8% copper-containing sample. Shanmugam et al. have also confirmed that as long as the input concentration of copper did not exceed 5%, the constituent phase of the nanopowders remained pure hydroxyapatite. Raising the copper concentration to 10% produced β-tricalcium phosphate as a secondary phase. In the same range, adding copper to hydoxyapatite reduced the crystal lattice size and crystal volume^22^. In a separate study, Stanic and colleagues discovered that when up to 4% copper was doped in hydroxyapatite, its crystallinity decreased from 86 to 77%, accompanied by a fall in network parameters^23^. Researchers have explained that when copper substitutes calcium, the smaller ionic radius of Cu^2+^ (0.73 Å) compared to Ca^2+^ (1.00 Å) causes apatite structural shrinkage.

Figure 1C shows FTIR data on as-dried powder samples. Peaks at 565, 588, and 603 cm^−1^ showed P-O-P bond bending^24^, while peaks at 1045 and 1090 cm^−1^ represented stretching^25^. Around 631 cm^−1^, hydroxyapatite's hydroxyl bond peak appeared^26^. Previous reports suggested that hydroxyapatite absorbed atmospheric moisture and carbonate. These absorptions were shown by peaks at 885, 1385, and 1430 cm^−1^ for absorbed carbonate 27 and 1630, 3450, and 3540 cm^−1^ for absorbed moisture^24,28^. Further, the disappearance of the NH_4_^+^ peak at 1422 cm^−1^, as well as the nitrate peaks at 1320 cm^−1^ and 1480 cm^−1^, indicated that these components were removed from the samples after several washes and dryings at 120 °C. The graph shows that copper doping did neither modify the location or intensity of pure HAp peaks nor create a new peak in the HAp structure.

Our results are comparable with those of Shu et al., who found that only hydroxyapatite-related peaks can be recognized in the FTIR graphs when copper incorporation varies from 0 to 8%. However, when the concentration hits 16%, the peaks associated with OH dissipate. At copper contents of 20% and 25%, new peaks appear at 729 and 938 cm^−1^, which the authors of the study attribute to the development of the CaCuP_2_O_7_ phase^21^.

Figure 1D displays the results of zeta potential analysis on the hydroxyapatite nanoparticles, indicating that elevating the copper content of the hydroxyapatite powders increased their zeta potential.

**Figure 2:**
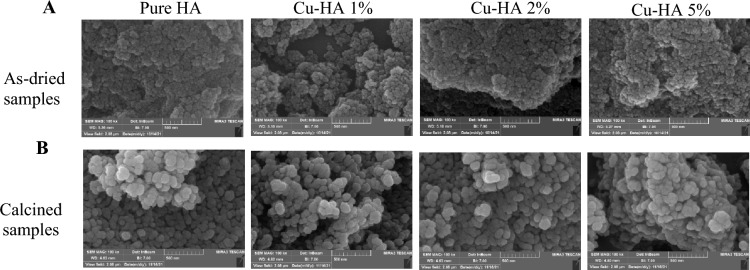
FESEM micrograph of as-dried (**A**) and calcined (**B**) powders.

Figures 2A,B show FESEM images of hydroxyapatite particles before and after calcination, respectively. As-dried hydroxyapatite particles were spherical and ranged from 15 to 70 nm in size, averaging 35–38 nm. Strong aggregation made nanopowder particles hard to differentiate. The photos also demonstrate that copper did not influence hydroxyapatite's shape or size distribution. Figure 2B again shows that the particles exhibited a spherical morphology, with sizes ranging from 40 to 140 nm and an average size between 70 and 80 nm. Nanoparticles are still found in agglomerates, but the intensity of agglomeration has decreased, making individual particles easier to detect. Like as-dried samples, copper did not influence particle shape, but as copper concentration rose, average particle size distribution slightly increased.

**Figure 3:**
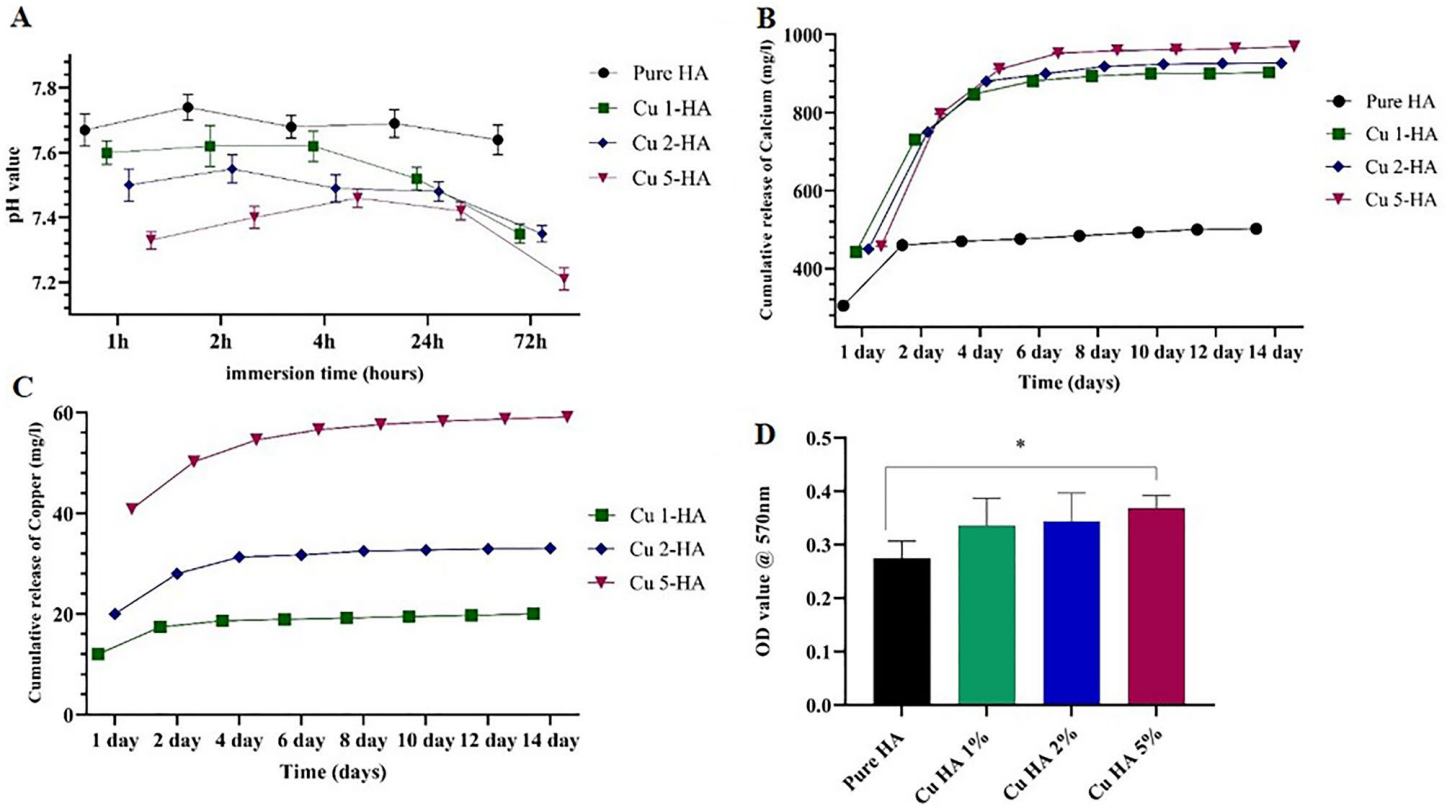
pH changes in the culture medium 72 h after powder addition (**A**). Concentration of calcium (**B**) and copper (**C**) ions liberated from powders up to 14 days after immersion in distilled water. Protein absorption on hydroxyapatite powders (**D**).

Figure 3A illustrates the pH variations in the culture medium over a period of 72 h following the addition of the powders. Overall, it can be observed that pure hydroxyapatite exhibited the greatest stability, as evidenced by the minimal pH fluctuation. Furthermore, the medium's pH declined in proportion to the copper level of the hydroxyapatite, indicating a correlation between the two variables.

To better understand powder degradation, we measured the amount of calcium and copper ions released from powders after immersing them in distilled water for several days.

Regarding calcium ion, pure HAp powders appear to release less calcium than other samples; one day after immersion in distilled water, the pure HAp sample released approximately 300 mg/liter of calcium, while the copper-containing samples released 450 mg/liter. This difference became more pronounced over time; by the end of the 14th day, the pure HAp sample had released a total of 500 mg/liter of calcium, whereas the copper-containing samples had nearly quadrupled that quantity, reaching 1000 mg/liter. Interestingly, while there was a substantial difference in calcium release between the pure and copper-containing samples, there was little variation in calcium release among the various copper-containing samples themselves (Fig. 3B).

In terms of copper ion release, as anticipated, the Cu-HA 5% sample showed the highest release (Fig. 3C). This indicates that by manipulating the amount of copper ions, it is possible to engineer the structure of hydroxyapatite to accomplish the desired release. All samples exhibited the same pattern of copper release from the powder. On the first day, there was an initial burst of copper release, which was then gradually reduced over the succeeding days. Nevertheless, copper was still being released from the samples over the course of the entire test (Fig. 3C).

The enhanced solubility of substituted HAps over pure HAp is well recognized^29^. According to Shu et al., increasing copper doping in HA disturbs the HAP lattice, resulting in lattice strain^29^. Furthermore, studies have shown that copper doping in HA enhanced its surface area, which aided in HA dissolution^30,31^. Nevertheless, it is evident that the gradual decline in the release of calcium and copper is a consistent trend across all samples (Fig. 3B,C). This is most likely due to the liquid surrounding the solid surface being saturated, causing the release rate to slow.

The Bradford test findings showed that, while all of the powders demonstrated significant protein absorption, the presence of copper in the hydroxyapatite composition boosted its capacity for protein absorption (Fig. 3D). Shi et al. also found that copper doping in hydroxyapatite improved BSA adsorption^32^. Copper-doped hydroxyapatite's positive charge may help adsorb negatively charged proteins like BSA and β-globulins from FBS^33^^.^ Our results also showed that copper doping raised the zeta potential of hydroxyapatite (Fig. 1D). Zheng et al. came to the same conclusion: by introducing Cu into the structure of mesoporous bioactive glass nanoparticles, the particles' zeta potential values rose, as did protein adsorption^34^. Stable coordination complexes may also form between copper ions on the hydroxyapatite surface and protein amino acid residues. It is important to remember that protein adsorption is a complicated process that depends on several factors, including the biomaterial's surface characteristics, chemical composition, and charge distribution, as well as solution parameters like pH and temperature. Further, reactive hydroxyapatite surfaces cause biomineralization and surface degradation, complicating protein adsorption^34^. More research is needed to understand how Cu inclusion affects protein adsorption.

### The antibacterial activity

**Figure 4:**
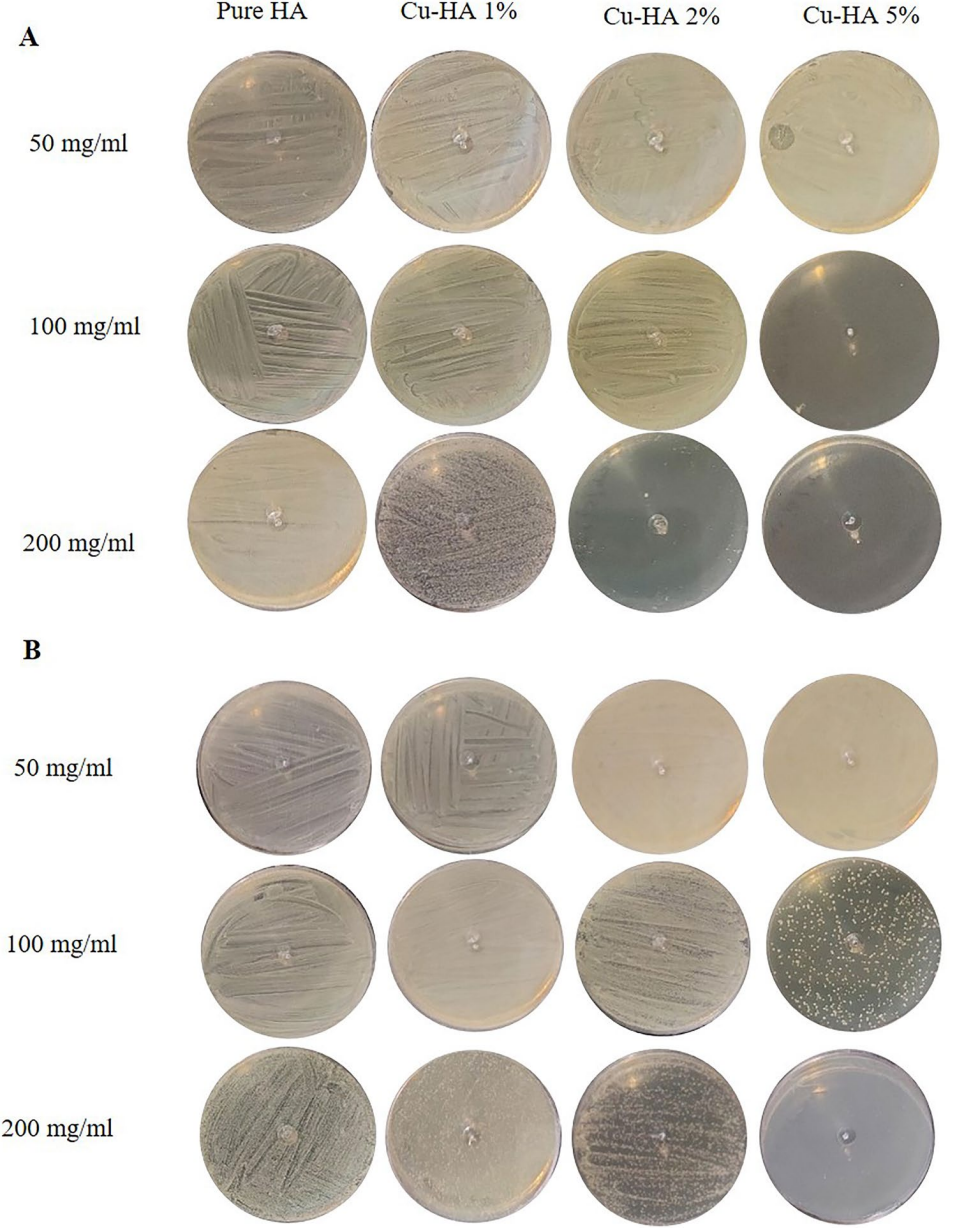
The anti-bacterial property of powders against E. coli (**A**) and S. aureus (**B**), after 24 h of cultivation with varied concentrations of copper-doped hydroxyapatite particles.

Figure 4A,B exhibit the antibacterial properties of hydroxyapatite nanoparticles. Figure 4A shows that 50 mg/ml hydroxyapatite, regardless of copper level, did not affect E. coli proliferation. However, 100 mg/ml of Cu-HA 5% reduced E. coli growth, leaving no discernible growth on the agar plate. After increasing the powder concentration to 200 mg/ml, Cu-HA 2% completely inhibited bacterial growth, similar to Cu-HA 5% apatite. Another salient observation in this row was that hydroxyapatite doped with 1% copper was substantially more effective than pure hydroxyapatite at inhibiting bacterial growth.

Similar to E. coli bacteria, the growth of S. aureus was not considerably affected by the presence of 50 mg/ml of apatite with any amount of copper (Fig. 4B). However, the second row of the figure demonstrated that the presence of 100 mg/ml of Cu-HA 5% severely inhibited bacterial growth. Despite this inhibition, bacterial growth was not entirely stopped. Finally, the third row of the figure demonstrates that practically all bacteria were killed in the presence of 200 mg/ml of Cu-HA 5%, and the presence of Cu-HA 2% considerably impeded bacterial growth. Even the maximum concentration of the pure HA or Cu-HA 1% nanoparticles did not stop bacterial growth.

When the antibacterial test findings in Fig. 4A,B are compared, it is obvious that S. aureus bacteria are more resistant to copper-containing hydroxyapatite nanoparticles. Cu-HA 2% failed to entirely stop the development of S. aureus bacteria at any concentration; however, it completely stopped the growth of E. coli at a concentration of 200 mg/ml. In addition, the development of E. coli was fully inhibited by Cu-HA 5% at a concentration of 100 mg/ml, whereas the inhibition of S. aureus growth necessitated a higher concentration of 200 mg/ml of the aforementioned powder.

These findings are consistent with previous research. Stanic and colleagues found that the gram-positive bacteria S. aureus were more resistant than E. coli because only E. coli has a growth-free zone surrounding the apatite disc^23^^.^ According to Najim et al.^18^ the membrane structure of S. aureus makes it less susceptible to metal-doped HA than E. coli. In contrast, Shu et al. discovered that 1 mg/ml hydroxyapatite inhibited S. aureus growth by 99%, whereas E. coli growth reduction was only 20%^21^.

Aside from the type of bacteria, copper-containing hydroxyapatite powder's bactericidal efficacy depends on its amount. Kim et al.^35^ found no antibacterial activities in 1 mg/ml of copper-doped hydroxyapatite powders. Stanic et al.^23^ found that copper-containing hydroxyapatite powders at 10 mg/ml had over 95% bactericidal activity, while Veljovic et al.^36^ found similar results at 10 mg/ml. Our findings differ dramatically from those of others in this regard. According to our observations, bacteria can thrive in the presence of 50 mg/ml copper-containing hydroxyapatite. To prevent their growth, the powder concentration should be increased to 100 or even 200 mg/ml. Variations in the final composition of copper-doped hydroxyapatite and the specific antibacterial test employed could explain these disparities. Nonetheless, more extensive research is required to acquire a thorough understanding of the antibacterial characteristics of copper-containing hydroxyapatite.

### The cell culture studies

#### Cell viability

**Figure 5:**
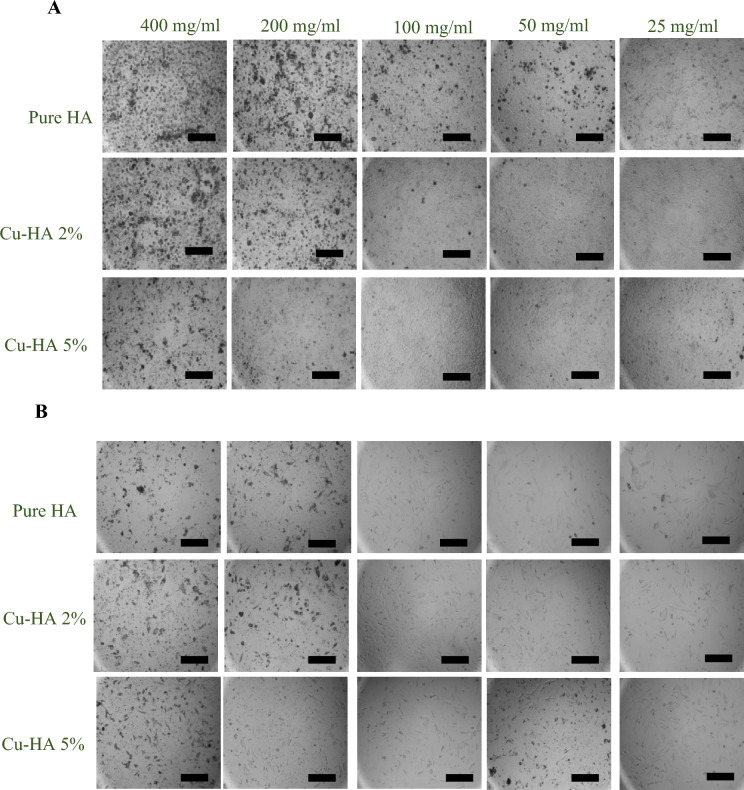
The cell morphology of rMSCs (**A**) and HUVECs (**B**) after being exposed to extracts from various powders for 72 h. The scale bar is equal to 300 µm.

Figure 5A,B depict the MSCs and HUVECs morphologies three days following exposure to hydroxyapatite powder extracts, respectively. As seen in the photos, the most concentrated form of pure hydroxyapatite or Cu-HA 2% extract (400 mg/ml powder in the culture medium) contained many dead cells. However, when exposed to Cu-HA 5%, the cell's tolerance improved. When the concentration of the extracts was reduced by half, the number of viable cells rose, and when the concentration of the extracts reached 100 mg/ml, the majority of cells were alive.

Our previous research indicated that copper at concentrations greater than 2.5 mM possessed antibacterial properties but was also toxic to eukaryote cells^12^. However, this study found different results. Bacteria were eradicated when exposed to 200 mg/ml of copper-containing hydroxyapatite powders, but the cells were more resistant. It is worth noting that in the preceding article, we used copper-containing culture media produced by dissolving CuSO_4_ in serum-free culture medium. Instead, in the current study, we used an ionic extract of hydroxyapatite, which comprises copper, calcium, and phosphorus. Calcium and phosphorus may help copper detoxify and minimize its harmful effects on eukaryotic cells. However, the results of multiple investigations indicate that cytotoxicity occurs when the concentration of copper released from ceramics exceeds a specific threshold^13,37,38^.

#### Osteogenic differentiation

**Figure 6:**
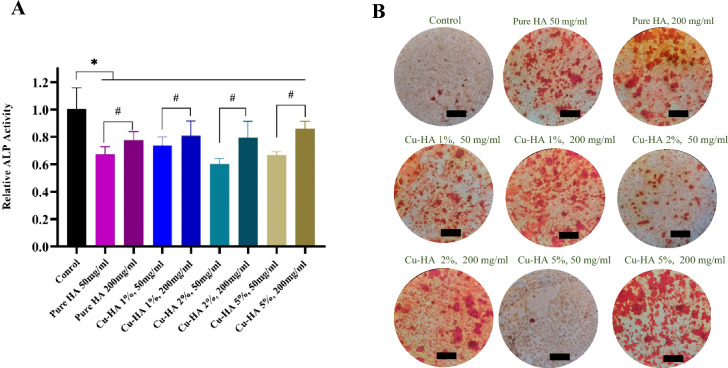
Osteogenic differentiation of mesenchymal stem cells, following exposure to coper-doped hydroxyapatite powder extracts. ALP activity (**A**) after 7 days exposure to extracts, all data were reported as means ± SD. *p < *0.05 when* compared to the control group and ^#^*p* < *0.05 when* the dilute and concentrated groups were compared (*P* < 0.05) . Alizarin red staining (**B**) on calcium deposited by stem cells 14 days after exposure to various powder extracts. The scale bar is equal to 300 µm.

ALP assay findings for rMSCs treated with hydroxyapatite powder extracts are shown in Fig. 6A. The level of ALP activity in all of the treated samples was lower than in the untreated control sample (*p* < 0.05). Among the four hydroxyapatite samples, there was a significant difference within each group between the 50 mg/ml and 200 mg/ml concentrations, so the more concentrated sample exhibited greater ALP activity (*p* < 0.05). No statistically significant difference existed across groups.

In Fig. 6B, rMSCs were stained with alizarin red after 14 days of culture with various extracts. Even without treatment, control sample cells deposited some calcium. Nonetheless, cells treated with hydroxyapatite extracts exhibited higher levels of calcium deposition. Notably, as the extract concentration increased to 200 mg/ml, calcium precipitate increased as well.

According to our data, copper did not significantly improve hydroxyapatite osteoconductivity, but it also did not have a detrimental impact on it. Some studies showed that raising copper concentrations within a safe range can enhance bone formation. Wu and coworkers, for example, doped 1, 3, and 5 mol percent copper into bioactive glass and found that human bone marrow stromal cells cultivated on glass with 5% copper exhibited the highest ALP activity and expression of OPN and OCN, which represent stem cell osteogenic differentiation^13^. Similarly, Wang and his colleagues doped 0.5, 1, and 3% CuO to the borate glass structure and found that the sample containing 3% copper has the highest ALP activity in bone marrow stem cells. Next, this concentration was selected as the optimal concentration, and after implantation in the rat occipital bone, it resulted in the development of more robust bone compared to the copper-free sample^39^. Qiang Wu et al. added 0.5%, 1%, and 1.5% copper to the glass framework and cultured MC3T3-E1 cells in the presence of the particle extracts. ALP tests and alizarin red staining showed that the 1% copper sample performed best^40^. Overall, these sets of investigations appear to agree on the fact that copper doping in the bioceramic structure plays a beneficial role in ossification.

Unlike the preceding studies, another group of researchers suggested that copper may not benefit bone precursor cell differentiation. Tian et al., for instance, subjected MC3T3-E1 cells to extracts of CaCuSi_4_O_10_ powders and observed a decrease in ALP activity after 7 days of cultivation compared to the control sample^37^. Notably, the authors relied exclusively on ALP activity, a measure of osteogenesis, which may not provide a thorough comprehension of the impact of copper on bone formation. Lin and colleagues discovered that doping of 0.4 and 0.8% copper in the bioactive glass composition elevated ALP activity in MC3T3-E1 cells. However, the scaffolds' ability to promote ossification was not significantly higher than control coper-free scaffolds when implanted in the parietal bones of mice for 6 weeks^41^. Another study found that copper-containing hydroxyapatite reduced OPN and RUNX2 protein expression compared to hydroxyapatite devoid of copper; however, this reduction was only noticeable when the copper concentration reached a high level of 5.3% by weight^42^.

Several variables contribute to these varying outcomes. First, the degradation rates and ionic release of bioceramics can vary, influencing the final outcome. Additionally, different research studies have employed distinct cell lines and strategies to determine osteogenic differentiation. This can lead to inconsistencies in the reported effects of copper.

#### The cell migration assay

**Figure 7:**
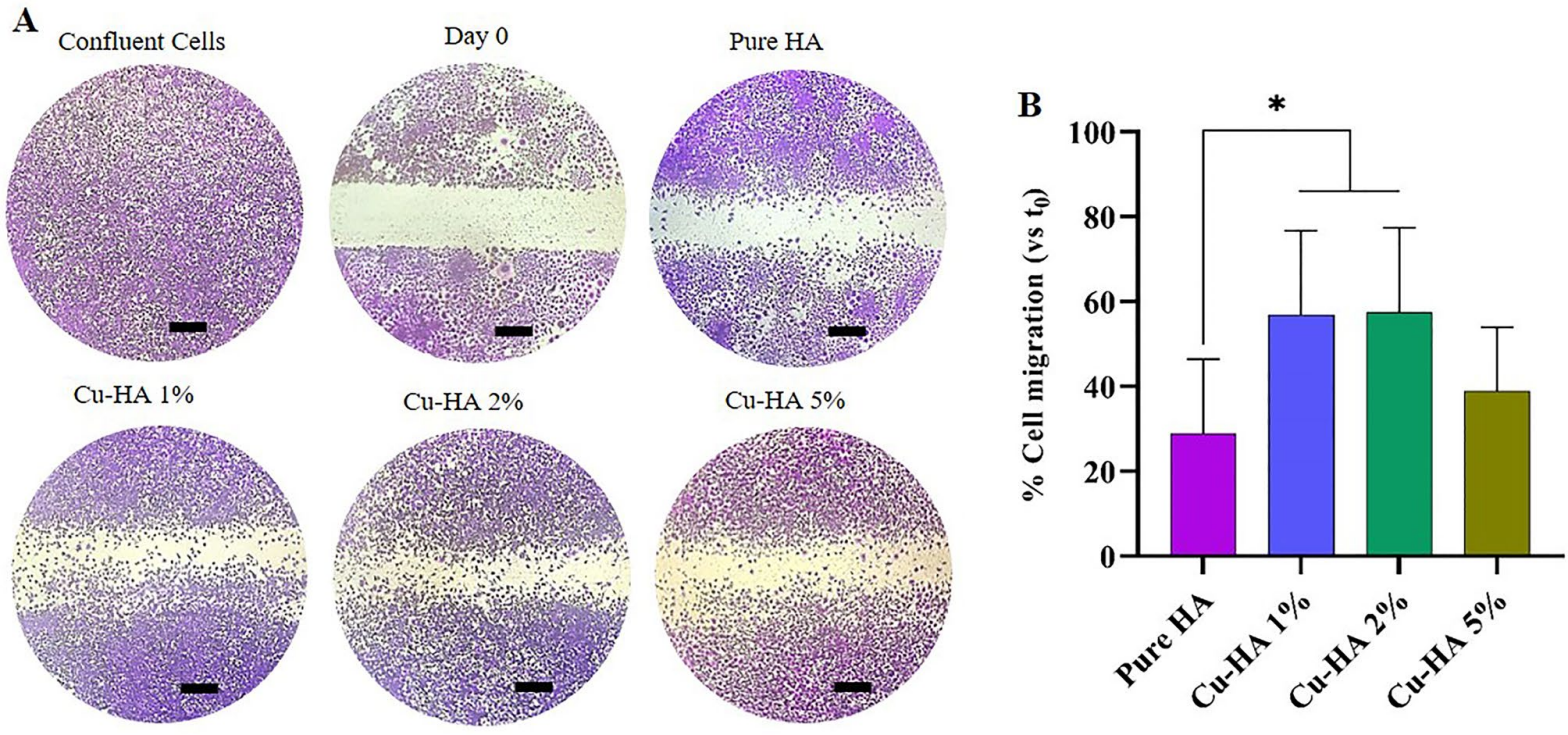
HUVECs migration assay: the cells subjected to different treatments, filled damaged region at diverse rates. Images were acquired using an optical microscope at the beginning of the experiment and 24 h later (**A**), and the relative injured width was charted during the cell migration assay at the beginning and 24 h (**B**). *p < 0.05 versus extract of pure hydroxyapatite. The scale bar represents 100 µm.

Figure 7 shows how hydroxyapatite extracts affect HUVEC migration. The defective area in all groups exhibited a gradual process of being filled. Notably, cell migration was accelerated when exposed to extracts derived from copper-containing powders, particularly in the Cu-HA 1% and Cu-HA 2% samples, where the observed augmentation reached a statistically significant level.

The earlier investigations also observed that copper doping in bioactive ceramics improved defect closure rates. For instance, Zhao et al. showed that the addition of copper to borate bioactive glass microfibers led to a significant increase in the migration of HUVECs. Further, the amount of copper doped in the bioactive glass had a positive correlation with the degree of this enhancement^43^. Zhou et al. also performed scratch assays and discovered that wound closure rates of HUVECs treated with conditioned medium obtained from copper-doped mesoporous bioactive glass (Cu-MBG) extract-primed BMSCs were significantly higher than those of the HUVECs exposed to the conditioned medium derived from MBG extract-primed BMSCs. This study provided experimental evidence that the Cu-MBG material induced a hypoxia-like milieu to effectively modulate the interactions between host immune-endothelial cells and mesenchymal-endothelial cells, ultimately leading to improved host angiogenesis and immune responses^38^.

## Conclusion

The goal of this investigation was to substitute calcium in the hydroxyapatite structure with copper at molar percentages of 1%, 2%, and 5%. Nevertheless, XRF analysis revealed that only about 0.22%, 0.72%, and 1.85% of copper were actually substituted for calcium. Despite this, doping the hydroxyapatite with the same amount of copper resulted in some changes to the lattice parameters and crystallinity of the pure hydroxyapatite. The findings of our study indicate that the controlled adjustment of copper ion content in hydroxyapatite can effectively modulate the release of both calcium and copper ions. Nevertheless, it is crucial to acknowledge that there is a limited concentration range of hydroxyapatite that is lethal to bacteria without harming eukaryotes. According to Figs. 4 and 5, this value can be approximately 100 or 200 mg/ml for Cu-HA 5%. Our findings also revealed that, while copper did not considerably improve hydroxyapatite osteoconductivity, it did not have a negative impact on it either. On the other hand, the doping of copper promoted endothelial cell migration, which can be regarded as a key indicator of angiogenesis. In summary, our findings indicate that adding copper to hydroxyapatite improves its intrinsic biocompatibility while simultaneously inducing antibacterial and angiogenic characteristics.

## Data Availability

The datasets used in the present study are available from the corresponding author on reasonable request.
